# The Flipped Classroom Approach: A Review of Cognitive Styles and Academic Performances

**DOI:** 10.7759/cureus.63729

**Published:** 2024-07-03

**Authors:** Fahad Somaa

**Affiliations:** 1 Occupational Therapy Department, Faculty of Medical Rehabilitation Sciences, King AbdulAziz University, Jeddah, SAU

**Keywords:** field independence, field dependence, academic performance, cognitive style, flipped classroom

## Abstract

There has been a surge in the efforts to efficiently improve students' academic performance recently such that their depth of learning as well as their academic attainment is elevated. The identification of the needs and requirements of students is imperative for this to materialize. The classroom setting differs around the globe, with several factors affecting how teaching and learning are conducted. Versatile cognitive preferences among students also play a unique role in the way they acquire education. Active learning is a strategy that challenges the traditional teacher-centered paradigm. One such technique in active learning involves the "flipped classroom," also called "inverted classroom."

The flipped classroom has been introduced as a novel educational technique in numerous areas of learning and has proven to have a more favorable influence. The concept of the flipped classroom is relatively novel in this regard. The idea and application of flipped classrooms and their effect on the academic performance of students with different cognitive styles have been studied by many researchers. Didactic lectures usually take a back seat (as supportive videos) to facilitate student learning in this format. This review aims to examine the mechanisms through which the flipped classroom approach affects the two cognitive styles: field-dependent and field-independent. The online component of the flipped classroom favors the field-independent students and helps them perform better through an in-class session as compared to the field-dependent students. The review further discusses the benefits of the flipped classroom for both field-dependent and field-independent students.

## Introduction and background

Traditional teaching and learning methods are quite often challenged in the modern era. For decades, giving lectures in a lecture hall to students has been the widely accepted teaching paradigm, with more focus on the teachers instead of students who are expected to sit for hours and listen. Critiques have claimed that this method inculcates passivity among students and the retention of knowledge is very minimal [1]. However, an opportunity is available for practitioners and researchers of education to diversify the approaches, methods, and techniques in delivering knowledge to students as the present student generation is heavily exposed to technology. “Active learning” is one of the approaches that challenge the traditional paradigm centered on teachers. “Flipped classroom”, also known as “inverted classroom”, is one such approach to active learning. In various disciplines of learning, the flipped classroom has been introduced as a new pedagogical approach and found to exert a more positive impact [2]. Of late, the approach of the flipped classroom has received much attention since it was first introduced in 2011 [3]. The main aim of the flipped classroom approach is to optimize students' time spent with their teachers [4].

In a flipped classroom setup, the preparation for in-class meetings needs to be carried out in advance, and online lectures, often in the form of supportive videos, facilitate this preparation. Also, there is a demand for the involvement of students during the lectures through peer instructions and problem-solving [5]. The use of technology in a systematic way in a flipped classroom both before the class and during the class activities enables the implementation of various activities of learning and is considered a key feature of the flipped classroom [6]. The current data regarding student performance is inconsistent [7-11]. The students’ learning performance as measured by course grades, GPA, or standardized test scores has been proven to improve with the use of the flipped classroom approach. One advantage of this method is the improvement gained in the learning performance of students, a key indicator of quality education. The improvement in students’ learning performance in the flipped classroom approach can be mostly attributed to active learning strategies [12].

The method and acquisition of learning vary among students, and it is termed as cognitive or learning style. Different individuals may find different modes of study or instructions effective, and hence their learning styles also differ. Although much deliberation has been conducted regarding several models of learning styles, it is clear that during the process of learning, different strategies are adopted by students based on their preferred style of learning [1]. When the teaching methods are tailored according to the cognitive styles of the students, their learning performance is enhanced [13].

## Review

The model of a flipped classroom

The flipped classroom model involves a blend of traditional and digital learning where the responsibility to study the material, of course, falls on the students. The digital study material might include homework in the form of practice problems and video lectures, while the classroom activities include group-based, active activities of problem-solving that target the thinking skills of higher-order students [14,15]. This is better defined as follows: “The flipped approach inverts the traditional classroom model by introducing course concepts before class, allowing educators to use class time to guide each student through active, practical, innovative applications of the course principles” [16]. The learning difficulties faced by the students could be due to their passive role during the long hours of traditional lectures and one remedy recommended by researchers is active learning [17]. Therefore, the flipped classroom method provides an environment of active learning and a “set of pedagogical approaches that move most information-transmission teaching out of class; use class time for learning activities that are active and social; and require students to complete pre-and/or post-class activities to fully benefit from in-class work” [5].

Owen and Strayer first discussed the topic of the flipped classroom in their paper [18] and cited Baker’s [19] work on the flipped classroom and the work on inverted classrooms by Lage et al. [20]. The core concept of these approaches is to flip or invert the work carried out inside and outside of a classroom by moving the work in time and space. So the focus is moved from increasing autonomy, engagement, or centeredness among students. There are many ways in which the traditional approach to a classroom can be flipped. in one of the strategies, the students are directed by the teacher to screencast, video lecture, or vodcast as part of their homework so they can learn the key concepts of the topic being taught. In the classroom, based on the knowledge acquired by students through homework completion, they engage in activities concerned with problem-solving and the teacher facilitates the process [3]. When these activities are performed in groups, it creates small peer learners’ communities [21]. Another strategy that teachers might use in flipped classrooms is teaching 'just-in-time' so that teachers can tailor instructions according to the needs of the students, and these needs can be assessed by getting students to fill out web-based questions before the start of the class [22].

The question that arises here is why should course coordinators and teachers flip their courses and classes. The most arguably reasonable answer could be that educators have realized that the most productive time with students is the face-to-face time offered by the flipped classroom approach. However, to some extent, there was also a need for teachers to meet the ever-changing demands of the education field. Over several years, the number of students attending lectures has decreased in universities due to several reasons, including a change in the work habits of the students, a desire for the delivery of education in a flexible manner, and the availability of the internet, which has made high-quality information easier to gather [23]. It is suggested that educators can meet the needs of students with the help of a flipped classroom model. With the help of advances in technology, teachers can quickly produce informative presentations informally and then post them online. This provides students an opportunity to learn whenever they want at their convenience and also saves the unnecessary effort teachers have to put in repeating information to the students to the students again and again [24].

The flipped classroom has been described as an educational technique with two significant parts by Bishop and Verleger [14]. The first part consists of computer technology and its use, such as delivering lectures through videos and the second part consists of involving students in learning activities that are interactive [14]. While designing a lesson for a flipped classroom, there are four main components [25]. First, a restructuring of time and environment by the teachers in which the students will learn flexibly. This is carried out based on the needs and expectations of an individual student as well as a group of students [25]. Secondly, the contents need to be taught in detail by a teacher with an approach that is learner-centered and provides opportunities rich in learning and the activities should be able to reflect a particular culture of learning for the specific student group [25]. Third, the difficulty level of the educational content should be regularly checked by the teachers. Also, teachers should monitor the notes written by students along with students' progress. Teachers should put in their efforts to apply strategies that promote active learning so that the conceptual understanding of the students improves [25]. Fourth and final, the teacher is a professionally qualified educator capable of keeping track of students’ learning processes, providing immediate feedback, and assessing the output of the students [25]. 

Moreover, there are three phases of the flipped classroom in which it can be classified [26,27]. The first phase is known as pre-class learning preparation. In this phase, the students read the study material with the help of a learning platform available online at their own pace. This is followed by the second phase, known as in-class learning activities. In this phase, teachers and students participate together in learning activities by going through the study material in the form of presentations, debates, discussions, and simulations [4,28]. The aim objective of this strategy is to increase learning efficacy by enhancing the teaching quality; therefore, with the help of in-class activities, the understanding of students is reinforced and broadened [29-31]. The third and last phase is post-class learning consolidation. It helps in enhancing the outcomes of learning by reviewing the study material. It enables students to learn even outside the environment of their classrooms. Students have the opportunity to apply the concepts they have learned in the classroom, fostering collaboration between themselves and their peers [32]. Therefore, evidence suggests that learning in flipped classrooms significantly affects students in different fields [33].

The impact of a flipped classroom on the learning of students

The flipped classroom method is significantly more effective in comparison to the traditional classroom when it comes to improving the learning performance of students. When the students have unrestricted access to the study material, either in the form of video lectures recorded earlier or presentations, they have an opportunity to learn at a time and place most suitable for them and at their own pace. This could explain why students have a positive perception of the flipped classroom and why flipped classrooms are more effective than traditional classrooms [34]. Another factor affecting the students's learning in a flipped classroom could be the ease of access to the videos so that students can repetitively watch the videos to gain a better understanding of the given topic [34,35]. The opportunity for more active learning in class can also explain the improvement in the understanding of students related to the topic discussed. Peer interaction is also promoted through in-class activities such as group discussions [36]. Moreover, instructors also have more time and opportunity to provide students with feedback during the face-to-face sessions [36]. The learning performance of students in a flipped classroom increases because they have many opportunities to apply what they have learned [35,36].

Another thing that makes flipped classrooms effective is the quizzes taken before the start of face-to-face in-class sessions. With the help of quizzes, instructors can assess what students have learned from the material given beforehand. These quizzes help students recall what they have learned from the pre-recorded material and it has been considered for a long time that prior knowledge is an important factor affecting the learning performance of students [37,38]. Students can better understand the new information when they recall what they learned previously because they can connect the new information with something they have already learned. Moreover, the memory path that leads to the retrieval of prior information becomes stronger. Therefore, on the next occasion, retrieval of this information becomes easy for the student [39]. Additionally, an instructor can identify the misconceptions of the students about the pre-class material. If these misconceptions are not addressed, further learning by the students can be prevented. Instructors can address the misconceptions of the students by either reviewing the pre-recorded lectures or adjusting the in-class plans for teaching based on the performance of the students [36,40]. Learning the material before in-class sessions, recalling the information, and getting concepts in the right way all enhance the learning performance of the students in a flipped classroom.

Flipped classrooms and students’ academic achievement

Performance outcomes of learning are represented by academic achievement, indicating the level attained by students for specific learning goals [41] and demonstrating the competence of students in extracurricular activities as well [42]. In recent years, many researchers have studied the impact of the flipped classroom on the academic achievement of students. Zengin conducted a study to investigate the impact of the flipped classroom on academic achievement and to understand students' opinions about this model [43]. The study observed a two-fold increase in the academic achievement of students in a flipped classroom [43]. Moreover, the study also observed that the learning of students was also facilitated by this approach, along with the visualization of teaching mathematics. Permanent learning was also contributed by the flipped classroom approach [43]. Zhonggen and Wang investigated the effectiveness of a flipped classroom approach and reported a higher score in students who were taught in a flipped classroom environment [44]. Another study was conducted on nursing students to evaluate the effect of the flipped classroom on their academic achievement. Traditional methods were used to teach the control group, whereas the flipped classroom method was used to teach the experimental group. Higher academic achievement was observed in the experimental group in comparison to the control group [45].

Cognitive styles and students’ academic achievement

The procedure through which an individual processes the information and how this affects their performance represents their cognitive style [46]. It can be said that one attribute of individuals that distinguishes them and has been most studied is their cognitive style. The approach through which an individual relates to others and thinks, solves, learns, and perceives things is defined as cognitive style [47]. According to Curry, psychometric qualities have three categories influencing the learning process of an individual. These categories include information processing style, instructional preferences, and cognitive style [48]. This model, though, received much criticism later [49] but still provided an overarching model for learning processes. As per Curry’s argument, there is more stability in cognitive style and it possesses more significance when it comes to complex learning [50]. Although it is also likely that new strategies for learning may be developed or adopted by students when there is a difference between their favored cognitive style and the task assigned to them, the role cognitive styles play in the learning process cannot be looked over, especially in the case of the initial phases of the study (51). Cognitive style is a person’s inbuilt characteristic that is relatively fixed and preferred [48,51] and consistently, it differs among individuals in the way in which the information is processed and organized by them [52]. Values, attitudes, and social interactions are also influenced by cognitive styles. In teaching and learning method development, cognitive style is very important [53-55]. Hence, the academic achievement of students is impacted in a better way by the combined presence of cognitive style factors and learning and teaching methods.

The two most acknowledged cognitive styles are “field dependence (FD) and field independence (FI), which are widely used in education research [56]. FI is described as “an analytical, in contrast to a global, way of perceiving, which entails a tendency to experience items as discrete from their backgrounds and reflects the ability to overcome the influence of an embedding context” [57]. It can be said that the capability of some people to extract some parts separately from complex backgrounds is greater, and the FI style tends to be more present in such individuals. Whereas the FD style is preferred for those individuals who are not able to do this easily. Moreover, the ability of a person to perceive a relevant and particular factor or item in a ‘field’ of various distracting articles is FI. The term ‘field’ in general psychology could be perceptual or abstract about a set of feelings, ideas, or thoughts from which an individual perceives relevant subsets specific to that individual. People with the FI cognitive style perceive the items as unrelated or discrete to the 'field', whereas people with the FD cognitive style prefer to perceive the whole background [58].

Similarly, when it comes to learning strategies, the cognitive style of an individual tends to require specific behavior. For example, individuals with an FD cognitive style will construct an overall image of the subject field and will not consider the small details [59]. Learning strategies that are passive and global are adopted by FD individuals because format-structure influences them and they require salient cues [60]. In comparison, active and analytical learning is employed by FI individuals because they focus more on the small details than the overall image for information processing [61]. The impact of cognitive styles on students’ academic achievement has been explored to understand this occurrence. A study found that the overall performance of FI individuals was worse than that of their FD-corresponding individuals [62]. Moreover, FD individuals exhibited a positive attitude when it came to social interactions. Therefore, their willingness to collaborate is greater, and they exhibit teamwork attributes as well [63]. Furthermore, another study found that teams with an FD-cognitive style had more group discussions, supported each other, and were more active than their corresponding FI team [64]. 

The influence of cognitive styles on the learning performance of students in a flipped classroom

In a flipped classroom, there is a blend of traditional and digital learning. Mostly, the responsibility of learning course material lies on the student, such as watching videos of lectures or practicing problems at home. Whereas classroom activities are more concerned with group discussions involving an active problem-solving thinking process that triggers the thinking skill of higher order during the classroom sessions [14,15]. Moreover, the environment of learning also improves for students with different learning styles in a flipped classroom [20]. Students in a flipped classroom can take charge of their learning pace and master the learning process. This enables students with lower academic performance to obtain a better understanding of the subject matter through discussion in a classroom session, and at the same time, classroom sessions will not become a source of boredom for higher achievers [65]. A study found that the academic achievement of students becomes better in a flipped classroom and the final scores were significantly better for those students with the FI cognitive style in comparison to those with the FD cognitive style [66].

Another study evaluated the academic achievement of English as a Foreign Language students and also investigated the difference in the achievement of the FD and FI cognitive styles among students. The study found a significant impact of the flipped classroom approach on the writing outcomes of the students [67]. These results are similar to the findings of previous research [68-73]. The study also observed that writing achievement improved significantly in students with FI cognitive style when they were placed in a flipped classroom approach in comparison to their corresponding FD cognitive style classmates [67]. It could be due to difficulties faced by students with FD cognitive styles in making a perception of learning material when the teaching method is less structured. Such students require more detailed instructions, explicit descriptions, and guidance from external sources in comparison to students with FI cognitive styles to cover the study material [47].

In a flipped-classroom approach, students with FD cognitive style are not able to attain a balance with FI cognitive style students, especially in online learning, because such activities demand creativity from students and they should be self-sufficient in using proper strategies for learning to make a better understanding of the subject matter to the maximum. Such a situation is more advantageous for FI students because they are more active and self-reliant when it comes to exploring the study material by describing their learning strategies. FI students can also paraphrase the language of the study material into simple language that is easily understood by them. Furthermore, FI students possess the ability to reorganize information from a variety of online sources. They gather detailed information from different sources, like presentations, reading passages, or video lectures, and afterward, they reorganize the data by joining all the gathered information to make a better understanding of the topic. Thus, students with the FI cognitive style can grasp learning material provided online in a much better way than their corresponding classmates with the FD cognitive style [67].

On the other hand, students with a cognitive style of field dependence face many difficulties in properly formulating learning strategies to study online material. Their paraphrasing skills are weak; therefore, the original information remains in its original form. Nevertheless, such students are also not able to restructure the information from online learning sources. They feel pressured when gathering information from different sources, like presentations, reading passages, or video lectures, and often the collected information is disjointed. Besides, the learning progress of field-dependent students is also slow. Extra time is required by them to review the material several times to eliminate any doubt they may have developed. Research suggests that field-independent students benefit more from online learning activities in a flipped classroom compared to their field-dependent classmates [74].

However, the activities in the classroom provide equal opportunities for field-dependent and independent students. Both cognitive style students get the same opportunity to have their confusions cleared on the areas that are troublesome for them at the beginning of the in-class activities. The instructors typically facilitate these discussions. When it comes to resolving student confusion, the instructors prioritize FD students first, as they tend to encounter fewer issues during in-class sessions. This helps in decreasing the gap in the understanding of FD students after their independent study. This way benefits both cognitive styles to be more ready to put together arguments while discussing in groups in classroom activities [67]. The orientation of FD students is beneficial whereas for FI students, it is personal [75]. Different activities for learning are needed by each of the cognitive styles because of social traits and the flipped approach provides an opportunity to accommodate both traits. The flipped classroom approach provides a lot of Spare time in the classroom for knowledge application [76-78], and preferred situations for learning are provided by teachers for both cognitive styles [67].

## Conclusions

The flipped classroom model significantly enhances academic achievement by enabling students to engage with learning materials online at their own pace before class, leading to better preparedness and comprehension during in-class activities. This approach caters to diverse cognitive styles and learning strategies, facilitating personalized learning experiences and improving performance outcomes. Teachers benefit from more effective time management, enabling them to focus on addressing individual student needs and misconceptions during class. This individualized attention helps all students, including those with different cognitive styles, to achieve their full potential. Overall, the flipped classroom model offers a flexible, student-centered learning environment that improves academic success through tailored instructional design and increased student-teacher interaction.

## References

[1] Avdic A, Åkerblom L (2015). Flipped classroom and learning strategies. Acad Conf Publish.

[2] Prensky M (2011). A huge leap ahead for the classroom: true peer-to-peer learning, enhanced by technology. Edu Tech.

[3] Milman N (2012). The flipped classroom strategy: what is it and how can it best be used. Dist Learn.

[4] Tucker B (2024). The flipped classroom. Educ Next.

[5] Abeysekera L, Dawson P (2015). Motivation and cognitive load in the flipped classroom: definition, rationale and a call for research. High Educ Res Dev.

[6] Strayer JF (2012). How learning in an inverted classroom influences cooperation, innovation and task orientation. Learn Environ Res.

[7] Davies RS, Dean DL, Ball N (2013). Flipping the classroom and instructional technology integration in a college-level information systems spreadsheet course. Educ Technol Res Dev.

[8] Mason GS, Shuman TR, Cook KE (2013). Comparing the effectiveness of an inverted classroom to a traditional classroom in an upper-division engineering course. IEEE Trans Educ.

[9] McLaughlin JE, Griffin LM, Esserman DA (2013). Pharmacy student engagement, performance, and perception in a flipped satellite classroom. Am J Pharm Educ.

[10] Pierce R, Fox J (2012). Vodcasts and active-learning exercises in a "flipped classroom" model of a renal pharmacotherapy module. Am J Pharm Educ.

[11] Street SE, Gilliland KO, McNeil C, Royal K (2015). The flipped classroom improved medical student performance and satisfaction in a pre-clinical physiology course. Med Sci Educ.

[12] Leo J, Puzio K (2016). Flipped instruction in a high school science classroom. J Sci Educ Technol.

[13] Onyekuru BU (2015). Field dependence-field independence cognitive style gender, career choice and academic achievement of secondary school students in Emohua Local Government Area of Rivers State. J Educ Pract.

[14] Bishop J, Verleger M (2013). The flipped classroom: a survey of the research. ASEE Annu Conf Expo Proc.

[15] King C, Piotrowski C (2015). E-learning and flipped instruction integration in business education: a proposed pedagogical model. J Instr Pedagog.

[16] (2024). The Academy of Active Learning Arts and Sciences. https://aalasinternational.org/.

[17] Andrews TM, Leonard MJ, Colgrove CA, Kalinowski ST (2011). Active learning not associated with student learning in a random sample of college biology courses. CBE Life Sci Educ.

[18] Owens DT, Strayer JF (2007). The effects of the classroom flip on the learning environment: a comparison of learning activity in a traditional classroom and a flip classroom that used an intelligent tutoring system. Ohio Univ.

[19] Baker JW (2000). The “classroom flip”: using web course management tools to become the guide by the side. Sel Pap 11th Int Conf Coll Teach Learn.

[20] Lage MJ, Platt GJ, Treglia M (2000). Inverting the classroom: a gateway to creating an inclusive learning environment. J Econ Educ.

[21] Sweet M, Michaelsen LK (2012). Team-Based Learning in the Social Sciences and Humanities: Group Work that Works to Generate Critical Thinking and Engagement. https://www.routledge.com/Team-Based-Learning-in-the-Social-Sciences-and-Humanities-Group-Work-that-Works-to-Generate-Critical-Thinking-and-Engagement/Sweet-Michaelsen/p/book/9781579226107.

[22] Berrett D. How “Flipping (2024). How “flipping" the classroom can improve the traditional lecture. Chron High Educ.

[23] Strelan P, Osborn A, Palmer E (2020). The flipped classroom: a meta-analysis of effects on student performance across disciplines and education levels. Educ Res Rev.

[24] (2014). Flipped Learning: Gateway to Student Engagement. Int Soc Technol Educ [Internet.

[25] (2024). Definition of flipped learning. https://flippedlearning.org/definition-of-flipped-learning/.

[26] Kong SC (2014). Developing information literacy and critical thinking skills through domain knowledge learning in digital classrooms: an experience of practicing flipped classroom strategy. Comput Educ.

[27] Kong SC (2015). An experience of a three-year study on the development of critical thinking skills in flipped secondary classrooms with pedagogical and technological support. Comput Educ.

[28] Estes M, Liu JC, Ingram R (2024). A review of flipped classroom research, practice, and technologies. https://www.hetl.org/a-review-of-flipped-classroom-research-practice-and-technologies/.

[29] Baepler P, Walker JD, Driessen M (2014). It’s not about seat time: blending, flipping, and efficiency in active learning classrooms. Comput Educ.

[30] (2024). 6 expert tips for flipping the classroom. https://campustechnology.com/articles/2013/01/23/6-expert-tips-for-flipping-the-classroom.aspx.

[31] Sparks RJ (2013). Flipping the classroom: an empirical study examining student learning. J Learn High Educ.

[32] Warter-Perez NJ, Dong J (2012). Flipping the classroom: how to embed inquiry and design projects into a digital engineering lecture. Proceed 2012 ASEE PSW Sec Conf.

[33] Sergis S, Sampson D, Pelliccione L (2017). Investigating the impact of flipped classroom on students’ learning experiences: a self-determination theory approach. Comput Hum Behav.

[34] Giuliano CA, Moser LR (2016). Evaluation of a flipped drug literature evaluation course. Am J Pharm Educ.

[35] Cotta KI, Shah S, Almgren MM, Macías-Moriarity LZ, Mody V (2016). Effectiveness of flipped classroom instructional model in teaching pharmaceutical calculations. Curr Pharm Teach Learn.

[36] Galway LP, Corbett KK, Takaro TK, Tairyan K, Frank E (2014). A novel integration of online and flipped classroom instructional models in public health higher education. BMC Med Educ.

[37] Hailikari T, Katajavuori N, Lindblom-Ylanne S (2008). The relevance of prior knowledge in learning and instructional design. Am J Pharm Educ.

[38] Merrill MD (2002). First principles of instruction. Educ Technol Res Dev.

[39] Dirkx KJH, Kester L, Kirschner PA (2014). The testing effect for learning principles and procedures from texts. J Educ Res.

[40] Tune JD, Sturek M, Basile DP (2013). Flipped classroom model improves graduate student performance in cardiovascular, respiratory, and renal physiology. Adv Physiol Educ.

[41] Ali S, Haider Z, Munir F, Khan H, Ahmed A (2013). Factors contributing to the students academic performance: a case study of Islamia university sub-campus. Am J Educ Res.

[42] Steinmayr R, Meißner A, Weidinger AF, Wirthwein L (2024). Academic achievement. https://www.oxfordbibliographies.com/display/document/obo-9780199756810/obo-9780199756810-0108.xml.

[43] Zengin Y (2017). Investigating the use of the khan academy and mathematics software with a flipped classroom approach in mathematics teaching. J Educ Technol Soc.

[44] Zhonggen Y, Guifang W (2016). Academic achievements and satisfaction of the clicker-aided flipped business English writing class. Educ Technol Soc.

[45] Naing C, Whittaker MA, Aung HH, Chellappan DK, Riegelman A (2023). The effects of flipped classrooms to improve learning outcomes in undergraduate health professional education: a systematic review. Campbell Syst Rev.

[46] (2020). The Adult Learner: The Definitive Classic in Adult Education and Human Resource Development.

[47] Witkin HA, Moore CA, Goodenough D, Cox PW (1977). Field-dependent and field-independent cognitive styles and their educational implications. Rev Educ Res.

[48] Curry L (1983). An organization of learning styles theory and constructs. Doc Res.

[49] Cools E, Bellens K (2012). The onion model: Myth or reality in the field of individual differences psychology?. Learn Individ Differ.

[50] (2004). Learning Styles and Pedagogy in Post-16 Learning: A Systematic and Critical Review. https://www.leerbeleving.nl/wp-content/uploads/2011/09/learning-styles.pdf.

[51] Roberts A (2006). Cognitive styles and student progression in architectural design education. Des Stud.

[52] Cools E, Van den Broeck H (2007). Development and validation of the cognitive style indicator. J Psychol.

[53] Wu H (2018). The effects of field independent/field dependent cognitive styles on incidental vocabulary acquisition under reading task. Theo Pract Lang Stu.

[54] Rogers ML, Tucker RP, Law KC (2019). The relationship between negative cognitive styles and lifetime suicide attempts is indirect through lifetime acute suicidal affective disturbance symptoms. Cogn Ther Res.

[55] Nawangwulan OR (2018). The effect of implementing podcast in enhancing students’ speaking achievement in the fully digital era. Int J Soc Sci.

[56] (2013). McKeachie’s Teaching Tips. https://books.google.co.in/books/about/McKeachie_s_Teaching_Tips.html?id=MWiC1zQaBpQC&redir_esc=y.

[57] (2002). An Introduction to Foreign Language Learning and Teaching. Beijing: Foreign Language Teaching and Research Press.

[58] Brown HD (2002). Principals of Language Learning and Teaching. Teaching and Research Press.

[59] Tekinarslan E (2009). Turkish university students’ perceptions of the world wide web as a learning tool: An investigation based on gender, socio-economic background, and web experience. Int Rev Res Open Distance Learn.

[60] Chen SC, Yang SJH, Hsiao CC (2016). Exploring student perceptions, learning outcome and gender differences in a flipped mathematics course. Br J Educ Technol.

[61] Wang HC (2014). Seeing the forest and the trees: pictorial complexity, field dependence/independence, and English Listening comprehension. English Teach Learn.

[62] Martínez CH (2004). Cognitive style in the dimension of field dependence-independence of university students from the Manizales area. Universitat Autònoma de Barcelona.

[63] Cakula S, Sedleniece M (2013). Development of a personalized e-learning model using methods of ontology. Procedia Comp Sci.

[64] Islam A, Islam S, Amin E (2022). Assessment of poultry rearing practices and risk factors of H5N1 and H9N2 virus circulating among backyard chickens and ducks in rural communities. PLoS One.

[65] Fulton KP (2012). 10 reasons to flip. Phi Delta Kappan.

[66] Chen YT, Liou S, Chen LF (2019). The relationships among gender, cognitive styles, learning strategies, and learning performance in the flipped classroom. Int J Human Comput Interact.

[67] Mubarok AF, Cahyono BY, Astuti UP (2019). Effect of flipped classroom model on Indonesian EFL students’ writing achievement across cognitive styles. Din Ilmu.

[68] Farah M (2024). The impact of using flipped classroom instruction on the writing performance of twelfth grade female Emirati students in the applied technology high school (ATHS). https://bspace.buid.ac.ae/items/04e2cb85-a5ea-47b3-8911-dcfaca489dce.

[69] Leis A, Tohei A, Cooke S (2015). The effects of flipped classrooms on English composition writing in an EFL environment. Int J Comput Assist Lang Learn Teach.

[70] Afrilyasanti R, Cahyono B, Astuti U (2017). Indonesian EFL students’ perceptions on the implementation of flipped classroom model Rida Afrilyasanti Sekolah Menengah Atas (senior high school) Negeri 8 at Malang, Indonesia. J Lang Teach Res.

[71] Ahmed M (2016). The effect of a flipping classroom on writing skill in English as a foreign language and students’ attitude towards flipping. US China Foreign Lang.

[72] Abdelrahman LAM, DeWitt D, Alias N, Rahman MNA (2017). Flipped learning for ESL writing in a Sudanese school. Turk Online J Educ Technol.

[73] Ekmekci E (2017). The flipped writing classroom in Turkish EFL context: a comparative study on a new model. Turk Online J Distance Educ.

[74] Laman JA, Brannon ML, Mena IB (2012). Classroom flip in a senior-level engineering course and comparison to previous version. 119th ASEE Annu Conf Expo.

[75] (2015). Understanding Second Language Acquisition. https://books.google.co.in/books/about/Understanding_Second_Language_Acquisitio.html?id=dZrICgAAQBAJ&redir_esc=y.

[76] Dickenson P (2015). Flipping the classroom in a teacher education course. Curriculum Design and Classroom Management.

[77] Prodoehl DE (2015). Flipping first-year English: Strengthening teacher-student conferencing through online modules. Implementation and Critical Assessment of the Flipped Classroom Experience.

[78] Çevikbaş M, Argün Z (2017). An innovative learning model in digital age: flipped classroom. J Educ Train Stud.

